# Influence of a Hyperglycemic Microenvironment on a Diabetic Versus Healthy Rat Vascular Endothelium Reveals Distinguishable Mechanistic and Phenotypic Responses

**DOI:** 10.3389/fphys.2019.00558

**Published:** 2019-05-10

**Authors:** Dhanush Haspula, Andrew K. Vallejos, Timothy M. Moore, Namrata Tomar, Ranjan K. Dash, Brian R. Hoffmann

**Affiliations:** ^1^Department of Biomedical Engineering, Medical College of Wisconsin, Marquette University, Milwaukee, WI, United States; ^2^Max McGee National Research Center, Children’s Research Institute, Milwaukee, WI, United States; ^3^Clinical and Translational Science Institute, Medical College of Wisconsin, Milwaukee, WI, United States; ^4^Department of Physiology, Medical College of Wisconsin, Milwaukee, WI, United States; ^5^Cardiovascular Center, Medical College of Wisconsin, Milwaukee, WI, United States; ^6^Center for Advancing Population Science, Medical College of Wisconsin, Milwaukee, WI, United States

**Keywords:** endothelium, hyperglycemia, diabetes, cardiovascular disease, mitochondrial function, oxidative stress

## Abstract

Hyperglycemia is a critical factor in the development of endothelial dysfunction in type 2 diabetes mellitus (T2DM). Whether hyperglycemic states result in a disruption of similar molecular mechanisms in endothelial cells under both diabetic and non-diabetic states, remains largely unknown. This study aimed to address this gap in knowledge through molecular and functional characterization of primary rat cardiac microvascular endothelial cells (RCMVECs) derived from the T2DM Goto-Kakizaki (GK) rat model in comparison to control Wistar-Kyoto (WKY) in response to a normal (NG) and hyperglycemic (HG) microenvironment. GK and WKY RCMVECs were cultured under NG (4.5 mM) and HG (25 mM) conditions for 3 weeks, followed by tandem mass spectrometry (MS/MS), qPCR, tube formation assay, microplate based fluorimetry, and mitochondrial respiration analyses. Following database matching and filtering (false discovery rate ≤ 5%, scan count ≥ 10), we identified a greater percentage of significantly altered proteins in GK (7.1%, HG versus NG), when compared to WKY (3.5%, HG versus NG) RCMVECs. Further stringent filters (log2ratio of > 2 or < –2, *p* < 0.05) followed by enrichment and pathway analyses of the MS/MS and quantitative PCR datasets (84 total genes screened), resulted in the identification of several molecular targets involved in angiogenic, redox and metabolic functions that were distinctively altered in GK as compared to WKY RCMVECs following HG exposure. While the expression of thirteen inflammatory and apoptotic genes were significantly increased in GK RCMVECs under HG conditions (*p* < 0.05), only 2 were significantly elevated in WKY RCMVECs under HG conditions. Several glycolytic enzymes were markedly reduced and pyruvate kinase activity was elevated in GK HG RCMVECs, while in mitochondrial respiratory chain activity was altered. Supporting this, TNFα and phorbol ester (PMA)-induced Reactive Oxygen Species (ROS) production were significantly enhanced in GK HG RCMVECs when compared to baseline levels (*p* < 0.05). Additionally, PMA mediated increase was the greatest in GK HG RCMVECs (*p* < 0.05). While HG caused reduction in tube formation assay parameters for WKY RCMVECs, GK RCMVECs exhibited impaired phenotypes under baseline conditions regardless of the glycemic microenvironment. We conclude that hyperglycemic microenvironment caused distinctive changes in the bioenergetics and REDOX pathways in the diabetic endothelium as compared to those observed in a healthy endothelium.

## Introduction

Cardiovascular disease (CVD) is one of the most significant pathophysiological phenotypes associated with the increase of diabetes worldwide. According to the Centers for Disease Control and Prevention, an estimated 9.4% of the US population is diabetic and 33.9% of the adult US population is prediabetic (Centers for Disease Control and Prevention, 2017). The unprecedented rise in the number of newly diagnosed diabetes cases, and disproportionately higher CVD mortality in individuals with diabetes than those without (Centers for Disease Control and Prevention, 2014), are both alarming statistics. This spike in a multitude of CVD risk factors roughly parallels the rise in consumption of sugar-sweetened or artificially-sweetened beverages (Hu and Malik, 2010). Since endothelial cell dysfunction has been identified at very early stages of type 2 diabetes mellitus (T2DM), and even preceding several symptoms of T2DM (Caballero et al., 1999), understanding key molecular impairments in the vascular endothelial cell at all stages of disease development will aid in identifying pathophysiological mechanisms of T2DM, which can be used for biomarker and therapeutic development for those with prediabetes and diabetes.

Hyperglycemia and insulin resistance, both characteristic features of T2DM, are known to be causally linked to endothelial dysfunction (Kolluru et al., 2012). Hyperglycemia-induced vascular complications are well-known to be associated with extensive tissue and organ damage (Aronson and Rayfield, 2002; Campos, 2012); the latter being one of the foremost reasons for a decreased life expectancy in patients with diabetes (Kalofoutis et al., 2007). Hence, therapeutic strategies should be aimed at improving and preserving endothelial functions in pathological conditions. This requires a comprehensive characterization of the endothelial proteome and transcriptome under both physiological and pathological conditions. Previous work in our laboratory and others have shown that hyperglycemic conditions alone displayed significant reduction in endothelial functions (Tesfamariam et al., 1990; Popov, 2010; Prisco et al., 2014), Elevation in the levels of superoxide production, potentially resulting in pro-inflammatory states and altered expression of vasodilatory mediators, are deemed as essential mechanisms of hyperglycemia-induced vascular damage (Nishikawa et al., 2000; Giacco and Brownlee, 2010; Sogawa et al., 2010).

The Goto-KakiZaki (GK) rat, a well-established non-obese, polygenic model of T2DM bred on the Wistar genetic background, exhibits pathophysiological characteristics that resemble those observed in human patients with diabetes (Akash et al., 2013; Wang et al., 2013). Among those are hyperglycemia, insulin resistance, vascular endothelial dysfunction, pro-inflammatory states, and beta cell dysfunction (Akash et al., 2013). Although differences in endothelial functions have been reported by several research groups in GK when compared to Wistar rats (Kazuyama et al., 2009; Gupte et al., 2010), a comprehensive list of molecular differences and/or similarities, both at the protein and gene level, in endothelial cells has not been published. While proteomic studies have been conducted on GK skeletal muscle samples (Mullen and Ohlendieck, 2010), a large-scale proteomic study on endothelial cell preparations has not been reported.

**FIGURE 1:**
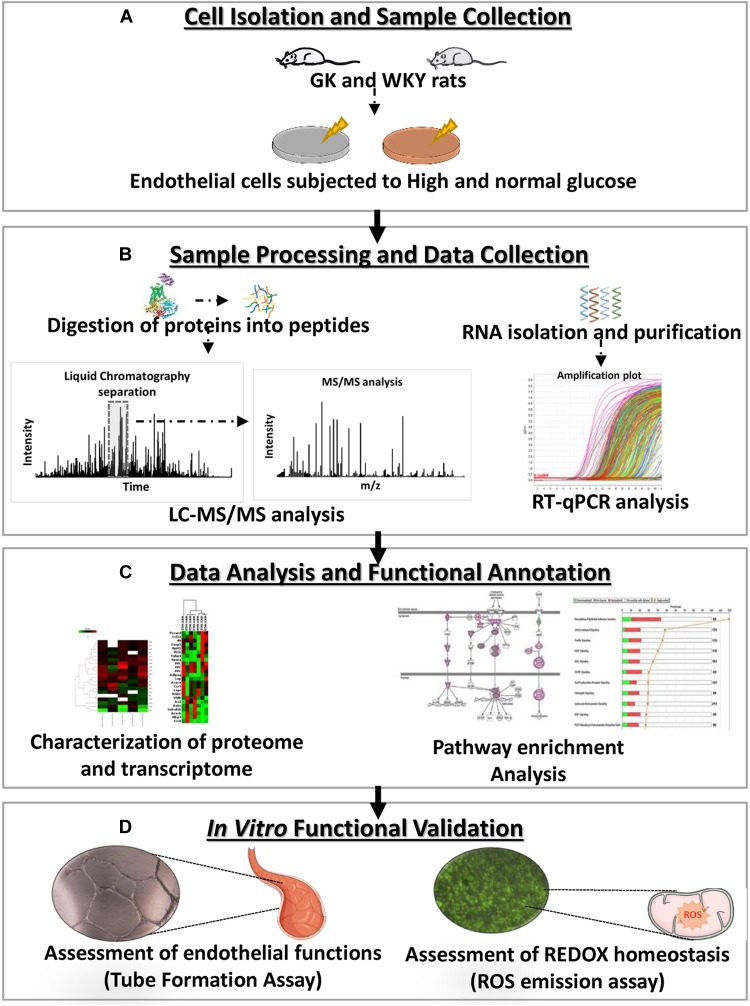
Experimental overview of the characterization, molecular analysis and functional validation of the influence of a hyperglycemic microenvironment on GK and WKY RCMVECs. **(A)** Primary RCMVECs were cultured from GK and WKY rats in endothelial cell media under normal (4.5 mM) or high glucose (25 mM) conditions for 2 weeks. **(B)** Protein samples were isolated and proteolytic peptides were subjected to liquid chromatography based separation, and subsequent tandem mass spectrometry analysis, followed by database identification. RT–PCR analysis was also performed to relate the proteomic dataset to key metabolic, inflammatory and apoptotic genes. **(C)** Ingenuity Pathway Analysis was employed to determine pathway enrichment and functional annotation of key target molecules. **(D)** *In vitro* assays were conducted for the assessment of angiogenic potential and redox states in GK and WKY RCMVECs under high glucose and normal glucose states.

While hyperglycemic state is an independent risk factor for endothelial dysfunction, a pro-inflammatory environment observed in diabetes is demonstrated to serve as a priming factor for hyperglycemia-induced vascular damage (Azcutia et al., 2010). Interestingly hyperglycemia has also been shown to further enhance the atherogenic effects of diabetic dyslipidemia (Renard et al., 2004; Funk et al., 2012). It remains largely unknown as in whether hyperglycemic states result in a disruption of similar cellular pathways under both a diabetic and non-diabetic background. The purpose of this study was to characterize the molecular alterations of a healthy and a dysfunctional vascular endothelium in response to a hyperglycemic environment. We hypothesize that under these different states the diabetic versus the healthy endothelium will display distinct molecular and functional differences, shedding insights on the response at different stages of disease development. To test this, we utilized high-throughput molecular analyses, proteomic and transcriptomic, in combination with *in vitro* functional testing for the identification of mechanisms of endothelial dysfunction in the control Wistar-Kyoto (WKY) and diabetes susceptible GK primary rat cardiac microvascular endothelial cells (RCMVECs) under hyperglycemic and normal conditions (Figure 1). Comparison of the hyperglycemia-induced changes in the proteome and transcriptome of GK RCMVECs with that of WKY RCMVECs, will allow us to understand the pathways that are critical under pathological conditions. Utilizing this approach, results of this study will help to delineate the molecular basis of endothelial dysfunction associated with genetic or environmental factors and to the genetic-environment interaction. An overview of the research design and methods are listed in Figure 1.

## Materials and Methods

### Animals and Primary Endothelial Cell Isolation

Rat models used for this study were handled according to protocols approved by the Medical College of Wisconsin (MCW) Institutional Animal Care and Use Committee. All rats were provided a normal chow diet (Purina) and water *ad libitum* while being housed and cared for at the Medical College of Wisconsin Biomedical Animal Resource Center. The inbred GK rats used to obtain cells for this study is a substrain of the Wistar rat. The inbred WKY rats used to obtain cells for the control in this study are also a substrain of the Wistar rat and this substrain was used because it exhibits a normotensive cardiovascular response, which was important to the comparisons in this study. At 14 weeks of age, GK and WKY rats were euthanized by CO_2_ inhalation, followed by thoracotomy according to approved protocols for endothelial cell isolation. In order to get sufficient primary endothelial cells from the isolations we had to use the hearts from two animals for each group. Hearts were removed from the euthanized rats, put in a separate 50 mL sterile tube with cold DMEM media containing 100 U/mL penicillin/streptomycin for each rat model separately. Intact heart tissue was then shipped overnight on ice to Cell Biologics. Primary cell isolations were then performed using the commercially available Cell Biologicals, Inc., primary cell isolation service to ensure proper collection and validation. Through this service all RCMVECs were grown in T75 tissue culture flasks pre-coated with a gelatin-based coating and expanded through passage 3 before cryo-preserving in vials containing 1 × 10^6^ cells/mL. Cell Biologicals uses a proprietary method of isolation involving PECAM-1 antibody purification and validated all cells for the expression of endothelial markers using PECAM-1 antibody (Cat no: sc-1506, Santa Cruz) or ZO-1 rabbit polyclonal antibody (Cat no: 617300, Life Technologies) by immunofluorescence. The isolated RCMVECs were also tested for uptake of the Dil-Ac-LDL (Cat no: L-35353, Invitrogen), a functional marker for endothelial cells. Following validation, all RCMVECs were snap-frozen, shipped to the laboratory at MCW, and were cryopreserved in liquid nitrogen long-term storage containers.

### Primary Cell Culture

Primary RCMVECs were expanded up to six to eight passages; at each of the early passages additional stocks of the cells were also cryo-preserved for future studies. All experiments were performed using RCMVECs that were matched for passages six to eight across experimental groups. RCMVECs were split at a ratio of 1:3 under the cell culture conditions specified by the Cell Biologics manufacturer protocol throughout the study. RCMVECs were cultured on 0.1% gelatin coated 100 mm dishes (Cat no: 430167, Corning) to confluence in basal MCDB131 endothelial cell media (Cat no: E3000-01B, US Biological) containing an EGM-MV supplement pack (Cat no: CC-4147, Lonza) plus or minus D-glucose supplementation (Cat no: G-8270, Sigma) according to previous protocols (Prisco et al., 2014). The basal media contains 4.5 mM D-glucose for normal conditions and this was supplemented with 20.5 mM D-glucose to bring the concentration to 25 mM in the high glucose media. For a hyperosmotic control to test the effect on the endothelial cells, a subset received media with 4.5 mM D-glucose supplemented with an additional 20.5 mM L-glucose (inactive form not utilized by the cells). Utilizing the tube formation assay as described below in the “Materials and Methods” section there were no observable differences in endothelial cell function (Supplementary Figure S1). As no differences were observed in endothelial function in this assay the remaining experiments were carried out without the presence of L-glucose to ensure this extra component did not influence the expression values measured in the study. Also, for each of the cell-based experimental tests the biological replicates (N) refer to cells that were cultured, passaged, and treated separately, whereas technical replicates refer to multiple tests run on the same biological replicate.

### Preparation of Samples for Mass Spectrometry Analysis

Rat cardiac microvascular endothelial cells were cultured to confluence in basal MCDB131 endothelial cell media (Cat no: E3000-01B, US Biological) containing an EGM-MV supplement pack (Cat no: CC-4147, Lonza) plus or minus normal high glucose (D-glucose; Cat no: G-8270, Sigma) (25 mM) as described above and according to previous protocols (Prisco et al., 2014). The basal media contains 4.5 mM glucose for normal conditions and this was supplemented with D-glucose to bring the concentration to 25 mM in the high glucose media. For this study there were four treatment groups: (1) WKY normal glucose treated RCMVECS (WKY NG), (2) WKY high glucose treated RCMVECS (WKY HG), (3) GK normal glucose treated RCMVECS (GK NG), and (4) GK high glucose treated RCMVECS (GK HG) over a three-week period. This time point was chosen as our previous study had shown significant phenotypic changes did not occur in RCMVECs under HG treatment prior to 2 weeks of treatment (Prisco et al., 2014). After 21 days of treatment with the appropriate media (passaging at 90% confluency), two RCMVEC plates per group (all matched between passage six-eight for the tandem mass spectrometry analysis) were washed three times with Dulbecco’s Phosphate Buffered Saline (DPBS; cat no: D8537, Sigma-Aldrich). Following the final DPBS wash, 1 mL of hypotonic lysis buffer (1 mol/L Tris 9 pH 7.5 and 100 mmol/L MgCl_2_) was added to each plate for cell scraping and the cell solution from both plates was added to a 15 mL Dounce homogenizer. Samples were diluted to 4 mL in hypotonic lysis buffer and mechanically lysed by twenty passages, followed by a 30 min water bath sonication. The lysate was then diluted in 6 mL membrane preparation buffer (280 mmol/L sucrose, 50 mmol/L MES (pH 6.5), 450 mmol/L NaCl, and 10 mmol/L MgCl_2_) and incubated on ice for 10 min. The sample groups were then centrifuged at 75,000 × *g* at 4°C for 2 h to pellet the membrane fraction. The supernatant was removed, concentrated and buffered swapped into 50 mM ammonium bicarbonate, pH 8.0 using Millipore Amicon Ultra15 3000 Da MWCO filters, and saved for cytosolic protein analysis. The membrane fraction was washed with 200 μL of membrane wash buffer (25 mmol/L NaCO3) in a thermomixer at 4°C for 30 min, brought to 6 mL with membrane preparation buffer for washing, and centrifuged an additional time as above. Supernatant from the membrane wash was removed and membrane fractions were resuspended in 50 mM ammonium bicarbonate, pH 8.0, 0.1% RapiGest (Waters), and 10 mM dithiolthreitol (DTT) for 60 min at 37°C in a thermomixer at 500 rpm; cytosolic fractions were also reduced in this manner. Samples were then alkylated with 20 mM iodoacetamide in the dark with slight agitation in a thermomixer at 300 rpm for 30 min at 23°C. Samples were then digested with a 1:50 sequence-grade Trypsin (Promega): protein sample ratio for 16 h at 37°C. Digested peptide mixtures were then desalted and concentrated via OMIX C18 zip-tips (OMIX) according to manufacturer protocol, as previously described (Hoffmann et al., 2013, 2017). Peptide concentrations were then measured using a Nanodrop, followed by fractionation and dilution to normalize concentrations across samples. Normalized fractions were then dried by vacuum centrifugation and stored at –20°C until tandem MS/MS analysis.

### Liquid Chromatography Tandem Mass Spectrometry (LC-MS/MS) Analysis

Dried peptide mixtures were resuspended in 8 μL of Orbitrap Buffer A (98% HPLC water/1.9% ACN/0.1% formic acid) to allow for three technical replicate injections of each biological replicate (each group has *N* = 3 and total runs/group = 9). Membrane and cytosolic fractions were run separate to limit dynamic exclusion and increase depth of sampling for each group, bringing it to a total of 18 total runs per group. For each run, tryptic peptide mixtures (1.9 μL) were run over an in-house 5 Å C18 resin (Phenomenex, Torrance, CA) packed pico-frit column (15 cm long, 50 μm inner diameter) coupled to a NanoAcquity Ultra performance liquid chromatography system (Waters, Milford, MA). A 240-min gradual gradient from Orbitrap Buffer A to Buffer B (98% ACN/1.9% HPLC water/0.1% formic acid) was applied to the peptide bound column. Eluted peptides were analyzed using a LTQ-Orbitrap Velos Mass Spectrometer (Thermo Fisher Scientific, Waltham, MA). Instrument settings and data dependent analysis were performed as indicated in our previous studies (Hoffmann et al., 2013, 2017; Kaczorowski et al., 2013; Karcher et al., 2015). Raw peptide mass spectra were searched against a UniProtKB Rodent Database through the SEQUEST and MASCOT search algorithms, followed by a search combine to keep only the top spectra match for each scan to avoid redundancies. Search parameters included a variable modification of +57-kDa for alkylation of cysteine and +16-Da for oxidation of methionine. In-house Visualize software was then utilized to filter samples data by removing redundancies, removing common contaminants, and applying a false discovery rate less than 5% (*P*-score > 0.85) (Halligan and Greene, 2011). Runs from each group (membrane and cytosolic) were then combined in Visualize, followed by the generation of comparison files between appropriate groups that were exported to Excel for further filtering. All comparison groups are run through a normalized data distribution *G*-test, considering total run counts, and subject to a stringent Benjamini–Hochberg *p*-value adjustment.

### Statistical Analysis of MS/MS Comparisons

In-house, open source Visualize software with built in statistical analysis for large proteomic dataset comparisons was used to compile all MS/MS data from the database searches (Halligan and Greene, 2011). Visualize uses a log-likelihood ratio *G*-test approximated through a chi-squared distribution with a single degree of freedom to determine significance (*p* < 0.05) as described in detail previously (Halligan and Greene, 2011; Hoffmann et al., 2017), followed by a stringent Benjamini–Hochberg *p*-value adjustment. Further stringent filters were applied to the comparison files, including a scan count > 10 in either group, a peptide count ≥ 2 in either groups, a log2ratio ≥ 2 or ≤ –2, and a *p*-value < 0.05 for the determination of significant and differentially regulated proteins (Tables 1–3). Heatmaps were constructed using PermutMatrix (Caraux and Pinloche, 2005). Uniquely altered proteins were also included in heatmap comparison. This was done by calculating the fold change for uniquely altered proteins by assigning an arbitrary value of 1 for the group that they were undetectable in.

**Table 1:** Selected differential protein expression targets important for endothelial function as measured by tandem LC-MS/MS analysis of GK NG versus WKY NG treated RCMVECs.

Accession	Description	WKY NG Peptides/Scans	GK NG Peptides/Scans	Normalized Log2ratio	*p*-value
P17244	Glyceraldehyde-3-phosphate dehydrogenase	0/0	3/185	GK NG only	1.72E-48
P00159	Cytochrome b.	0/0	3/53	GK NG only	2.78E-12
O35763	Moesin	0/0	36/95	GK NG only	7.67E-24
P31000	Vimentin	0/0	32/146	GK NG only	8.06E-38
Q3KRE8	Tubulin beta-2B chain	0/0	13/265	GK NG only	2.38E-70
P05503	Cytochrome c oxidase subunit 1	2/30	1/2	3.71	0.003
P82471	Guanine nucleotide-binding protein G subunit alpha	5/27	2/4	–2.95	0.01
Q9JM53	Programmed cell death protein 8	11/33	1/3	–3.66	5.44E-05
Q91YT0	NADH-ubiquinone oxidoreductase	8/23	1/1	–4.73	0.001
Q9DCJ5	NADH dehydrogenase 1 alpha subcomplex subunit 8	3/16	0/0	WKY NG only	0.005


*All proteins indicated had to meet the following stringency criteria for one of the groups in the comparison (Scan count ≥ 10 for atleast one group, Normalized Log2ratio ≥ 2 or ≤ –2, *p-*value < 0.05) (*N* = 3; 9 technical replicates). The complete comparison dataset can be found in Supplementary Table S1.*

**Table 2:** Selected differential protein expression targets important for endothelial function as measured by tandem LC-MS/MS analysis of WKY HG versus WKY NG treated RCMVECs.

Accession	Description	WKY NG Peptides/Scans	WKY HG Peptides/Scans	Normalized Log2ratio	*p*-value
P42225	Signal transducer and activator of transcription 1	1/1	4/23	4.33	0.02
P42123	L-lactate dehydrogenase	3/15	12/102	2.57	3.34E-11
P14942	Glutathione S-transferase alpha-4	3/6	4/60	3.13	2.1E-07
P21708	Extracellularsignal-regulated kinase 1	4/9	8/81	2.98	3.25E-10
P53534	Glycogen phosphorylase	6/11	16/58	2.21	5.23E-4
Q9ESW4	Acylglycerol kinase	2/3	4/34	3.31	0.002
Q8C675	Glucose-6-phosphate isomerase	7/61	6/17	-2.03	9.88E-05
Q62470	Integrin alpha-3 precursor	5/35	3/7	-2.51	0.007
Q8C0L0	Thioredoxin domain-containing protein 13 precursor.	3/23	1/2	-3.71	0.0097
P27274	CD59 glycoprotein precursor	3/51	2/4	-3.86	1.64E-09
Q9JHY1	Junctional adhesion molecule A precursor	3/23	1/1	-4.71	0.001
Q99376	Transferrin receptor protein 1	11/79	3/10	-3.17	4.17E-13
P11608	ATP synthase protein 8	3/106	2/8	-3.92	1.37E-22
Q505J6	Mitochondrial glutamate carrier 2	2/25	6/1	-4.83	2.28E-04
P03899	NADH-ubiquinone oxidoreductase chain 3	1/17	0/0	WKY NG only	0.0030


*All proteins indicated had to meet the following stringency criteria for one of the groups in the comparison (Scan count ≥ 10 for atleast one group, Normalized Log2ratio ≥ 2 or ≤ –2, *p*-value < 0.05) (*N* = 3; 9 technical replicates). The complete comparison dataset can be found in Supplementary Table S2.*

**Table 3:** Selected differential protein expression targets important for endothelial function as measured by tandem LC-MS/MS analysis of GK HG versus GK NG treated RCMVECs.

Accession	Description	GK NG Peptides/Scans	GK HG Peptides/Scans	Normalized Log2ratio	*p*-value
O35457	Chemokine receptor CCR11	0/0	1/25	GK HG only	1.69E-05
Q6P0K8	Junction plakoglobin	3/8	13/148	4.28	8.3E-33
Q68FY0	Ubiquinol-cytochrome-c reductase complex core protein 1	3/20	15/353	4.21	2.87E-80
Q2PS20	Junctophilin-2	3/5	5/36	2.92	9.9E-04
P39052	Dynamin-2	2/9	4/41	2.26	0.009
P42123	L-lactate dehydrogenase B chain	10/19	9/83	2.19	7.2E-08
Q62745	CD81 antigen	2/50	2/199	2.06	2.75E-20
P07943	Aldose reductase	30/830	18/189	-2.06	6.15E-86
Q62470	Integrin alpha-3 precursor	5/64	5/11	-2.47	3.7E-06
P27274	CD59 glycoprotein precursor	4/52	1/4	-3.63	7.65E-08
P15429	Beta-enolase	10/327	1/16	-4.28	4.96E-70
Q9JHY1	Junctional adhesion molecule A precursor	5/42	1/1	-5.32	3.31E-08
Q35763	Moesin	36/95	0/0	GK NG only	1.54E-25
P31000	Vimentin	32/146	0/0	GK NG only	1.88E-40
P17244	Glyceraldehyde-3-phosphate dehydrogenase	3/185	0/0	GK NG only	7.92E-52


*All proteins indicated had to meet the following stringency criteria for one of the groups in the comparison (Scan count ≥ 10 for atleast one group, Normalized Log2ratio ≥ 2 or ≤ –2, *p-*value < 0.05) (*N* = 3; 9 technical replicates). The complete comparison dataset can be found in Supplementary Table S3.*

### Bioinformatics Analysis

In order to gain insight into the relevant signaling processes and biological functions, the significantly differential protein lists for each comparison were imported into Ingenuity Pathway Analysis (IPA; Ingenuity Systems)^1^. The stringency of the filters for IPA were set to include a *p*-value of < 0.05. The core analysis platform was selected for data analysis and interpretation. Ingenuity knowledge base was used as the reference dataset, and the proteins were matched both direct and indirect relationships. For the interaction networks, the default setting of 35 molecules per network and 25 networks per analysis, were employed. The software uses Fisher’s Exact Test to determine the magnitude of association between the proteins of the dataset and a disease or a pathway or a function (enrichment analysis). Since only the presence or absence of the protein is taken into account, it does not predict an increase or a decrease in the biological process. In order to predict the directional change of a cellular process, such as an activation or an inactivation of a cellular pathway, *z*-score is employed. *z*-Score measures the association between predicted and observed changes. A *z*-score of ≥ 2 indicates significant association and an increased activity. A *z*-score of ≤ –2 indicates significant association and a decreased activity. Benjamini–Hochberg correction is also employed by the software to account for the false discovery rate due to multiple comparisons.

### RNA Isolation, cDNA Conversion, and Quantitative RT–PCR (qPCR)

Total RNA was extracted from all RCMVEC groups (WKY NG, WKY HG, GK NG, GK HG) using the RNeasy Minikit (Cat no: 74104, Qiagen) according to the manufacturer protocol. Before this protocol is employed, all cell pellets were resuspended in 600 μL of RLT buffer, followed by mechanical lysis with multiple passes through a 23-gauge needle. Following the RNA isolation, the concentration and purity of the RNA preparations were assessed using a NanoDrop ND-1000. All samples had 260/280 and 260/230 ratios ≥ 2.0. cDNA was synthesized from 2 μg of total RNA by employing RT^2^ First strand Kit (Cat no: 330404, Qiagen) as per the manufacturer instructions. All cDNA samples were stored at –80°C until use for qPCR. In order to determine the implications of diabetes states and/or hyperglycemic conditions, we then performed real-time (RT)–qPCR analysis using the RT^2^ Profiler PCR Plate (Cat no: PARN-156Z, Qiagen) designed for the detection and analysis of eighty-four key metabolic, inflammatory and apoptotic genes of four separate groups at once. RT-qPCR was performed on cDNA samples from all the four groups on the Applied Biosystems^TM^ QuantStudio^TM^ 6 Flex Real-Time PCR System by using SYBR green detection master mix (Cat no: 330522, Qiagen).

### Analysis of RT-PCR Data

PCR data (*n* = 4 for each group) was uploaded in the online Qiagen data analysis software and normalized to β2 microglobulin, the housekeeping gene which showed consistency across all the runs and various comparisons groups on the qPCR plate. Any threshold cycle value (Ct) greater than 35 was considered to be negative for that particular gene. No genomic DNA was detected in any of the groups (Ct > 35 or undetectable). Fold change was calculated based on 2–ΔCt method (Livak and Schmittgen, 2001). Experimental comparisons were made between the test groups (WKY HG, GK HG) with their respective control groups (WKY NG and GK NG). The values were calculated based on a Student’s *t*-test. Further filters were applied, including a *p*-value of < 0.05 and log2ratio > 2 or < –2, to identify transcripts that were significantly altered in response to hyperglycemic as compared to normal glycemic treatments of GK and WKY RCMVECs.

### Tube Formation Analysis

Following a three-week treatment in normal (4.5 mM) and high glucose (25 mM) media, all RCMVEC groups (WKY NG, WKY HG, GK NG, GK HG) were subject to an a tube formation assay to measure endothelial functions according to previous protocols (Hoffmann et al., 2013, 2017; Prisco et al., 2014). RCMVECs were grown as described above, washed two times with DPBS, were lifted using 1 mL of 0.1% trypsin (Cat no: 15400054, Thermo Fisher Scientific) for 5 min at 37°C, diluted in 10 mL of MCDB131 EGM-MV media plus or minus high glucose, and pelleted by centrifugation at 300 × *g* for 5 min. The RCMVEC pellets were resuspended in 1 mL of MCDB131 EGM-MV media plus 10% FBS and counted using a cell countess system (Invitrogen). RCMVECs were diluted to a concentration of 20,000 cells per 1 mL of media for each well of a four-chamber slide (Cat no: 154534, Lab-Tek/Thermo Fisher Scientific) coated in 250 μL of Growth Factor Reduced Matrigel (Cat no: 354230, Corning). Chamber slides were incubated for 48 h at 37°C and image at 4× and/or 10× magnification using a TS100 Inverted Microscope (Nikon Corporation) for analysis of the mean tube length (μm), branch points, area (μm^2^), and thickness (μm) using open-access PipeLine tube formation analysis software (Prisco et al., 2014). The results were averaged across biological replicates (*n* = 3; with 8 technical replicates of each). Non-parametric Kruskal–Wallis one way ANOVA based on ranks was employed to determine if any differences exist between the median values for the various groups. This was followed by a multiple comparisons test (Dunn’s Method) to determine if any specific group differs from the others (SigmaPlot).

### Isolation of Enriched Mitochondria Fraction and Respiration Measurement

Functional mitochondria were isolated from GK and WKY RCMVECs, following a three-week high glucose treatment, using the Mitochondria Isolation Kit (Cat no: MITOISO2, Sigma-Aldrich) according to the manufacturer protocol. A total of 3 × 10^7^ cells were utilized for mitochondrial isolation from each of the 4 groups (WKY NG, WKY HG, GK NG, GK HG). Following trypsinization and PBS washing, the cell pellet was suspended in lysis buffer supplied with the isolation kit. Using a 23-gauge needle, the cells were homogenized. The homogenate was then centrifuged at 600 × *g* for 10 min at 4°C to remove cell debris. Pure mitochondria were then obtained by subjecting the supernatant to a second centrifugation step at 11,000 × *g* for 10 min at 4°C. The pellet was then suspended in 1× storage buffer from the kit for use in functional studies. Mitochondria protein content was measured using the BCA method as per the manufacturer’s instructions. The final pellet was suspended in CelLytic M cell lysis reagent for protein measurement. All reagents were prepared in 1× Extraction buffer containing protease inhibitor cocktail (1:100; (v/v). All isolation procedures were carried out on ice. Depending on the protein content, the suspension volume was adjusted to have a final protein concentration of 125 μg protein/mL in the O2K Core: Oxygraph-2k chamber (Cat no: 10000-02, Oroboros Instruments).

Mitochondrial respiration measurement was made using the OROBOROS Oxygraph-2k for high-resolution respirometry at 37°C with 2 mL volume of respiration buffer (130 mM KCl, 2.5 mM K2HPO4, 20 mM MOPS, 1 mM EGTA, and 0.1% BSA at pH 7.15 adjusted with KOH) in both chambers, with a stirring speed of 700 rpm. Prior to the start of the experiments, the chambers and the stoppers were washed with 70% ethanol. Mitochondrial experiments were run after achieving a stable oxygen flux signal. About 125 μg of mitochondria protein suspended in 1× storage buffer was loaded in the chamber. State II respiration was initiated by glutamate and malate (10 mM) as the respiratory substrates. Following the addition of substrates, ADP (250 μM) was added to stimulate state III respiration. State IV respiration was measured after phosphorylation of ADP to ATP. To test the functional integrity of mitochondria on a given experimental day, the respiratory control index (RCI) was calculated from the respiration rate ratio of state III/state II or state III/state IV. Isolated mitochondrial preparations with RCI > 3.5 were included for the experiments. The rate of oxygen consumption was averaged across biological replicates (*n* = 4–8 biological replicates). Non-parametric Kruskal–Wallis one way ANOVA based on ranks was employed to determine if any differences exist between the median values for the various groups. Multiple comparison test was not performed since there was not a statistically significant difference between various groups (SigmaPlot).

### Reactive Oxygen Species (ROS) Assay

Ensuing the completion of the three-week high glucose treatment, the cells were treated with tumor necrosis factor-alpha (TNF-α) (5 ng/ml) (Cat no: H8916, Sigma) or phorbol 12-myristate 13-acetate (PMA) (50 μM) (Cat no: 524400, Calbiochem) or menadione sodium bisulfite (100 μM) (Cat no: M5750, Sigma) for a duration of 1 h to induce stress. The cells were then incubated with CellROX green reagent (Cat no: C10444, Thermo Fisher Scientific) at a concentration of 10 μM for 30 min at 37°C, followed by 3 washes with PBS, and analysis (EX/EM 485/520 nm) using SoftMax^^®^^ Pro Microplate Data Acquisition and Analysis Software. The mean signal intensity was averaged across biological replicates (*N* = 3; with 4 technical replicates of each). One way ANOVA followed by Tukey test was employed to determine if any statistical differences exist between the various treatment groups (SigmaPlot).

### Pyruvate Kinase Metabolic (PKM) Assay

Pyruvate Kinase Metabolic activity was determined using the PKM activity assay kit as per the manufacturer’s protocol (Cat no: K709-100, Biovision). Briefly, two million cells were collected from each of the four groups, following the three-week high glucose treatment, and lysed with 100 μl of PKM assay buffer by mechanical lysis with multiple passes through a 23-gauge needle. Cell lysate was then centrifuged at 10000 × *g*. 10 μl of the cell lysate per well was then employed for the determination of PKM activity. Optical density was measured at 570 nm at two-minute intervals for 30 min using SoftMax^^®^^ Pro Microplate Data Acquisition and Analysis Software. The mean signal intensity was averaged across biological replicates (*N* = 3 biological replicates). PKM activity was calculated for two absorbance readings in the reaction linear range. Non-parametric Kruskal–Wallis one way ANOVA based on ranks was employed to determine if any differences exist between the median values for the various groups. This was followed by a multiple comparisons test (Dunn’s Method) to determine if any specific group differs from the others (SigmaPlot).

## Results

### Differential Protein Expression of the Vascular Endothelium in Response to Hyperglycemic Conditions and a Permissive Background for Diabetes

**FIGURE 2:**
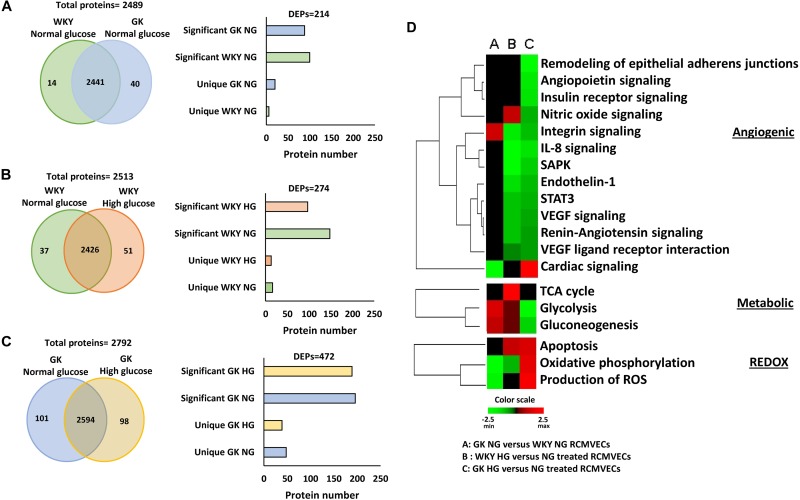
Proteomic comparisons of GK and WKY RCMVECs under hyperglycemic conditions versus normal glycemic conditions. Tandem LC-MS/MS analysis was employed for the measurement of proteins in WKY and GK RCMVECs under normal (NG; 4.5 mM) or high (HG; 25 mM) glucose conditions (*n* = 3, 9 technical replicates). The proteins were then subjected to stringent filters (log2ratio ≤ –2 or ≥ 2 and *p-*value < 0.05) for the identification of differentially expressed proteins (DEPs), which were further broken down into significantly elevated and uniquely expressed across the various groups. **(A)** Total proteins and filtered DEPS identified in GK NG versus WKY NG RCMVECs represented as a Venn diagram and histogram, respectively. **(B)** Total proteins and filtered DEPS identified in WKY HG versus NG treated RCMVECs represented as a Venn diagram and histogram, respectively. **(C)** Total proteins and filtered DEPS identified in GK HG versus NG treated RCMVECs represented as a Venn diagram and histogram, respectively. **(D)** Heatmaps were created based upon the IPA based *z*-score algorithm for significantly altered proteins to predict the activation state of biological processes implicated in endothelial function for GK NG versus WKY NG RCMVECs, GK RCMVECs (HG versus NG) and WKY RCMVECs (HG versus NG). Red indicates activation, and green indicates repression of functions.

Protein lysates isolated from primary RCMVECs derived from the control WKY and diabetes permissive GK rat models, under hyperglycemic and normal glycemic conditions, were subjected to LC-MS/MS analysis. Database matching and filtering (FDR ≤ 5%, scan count ≥ 10), resulted in the identification of 2489 proteins when GK NG was compared to WKY NG (Figure 2A). Only 54 out of 2489 proteins (2.2%) were uniquely present in either groups under baseline conditions (NG). This is suggestive of a low percentage of dissimilar proteins in GK NG when compared to WKY NG RCMVECs. By employing similar filter criteria (FDR ≤ 5%, scan count ≥ 10), 2514 proteins were identified in WKY HG versus NG treated RCMVECs, and 2793 proteins in GK HG versus NG RCMVECs. While 199 out of 2792 proteins (7.1%) were uniquely altered in GK HG versus NG treated RCMVECs (Figure 2C), only 88 out of 2513 proteins (3.5%) were uniquely altered in WKY HG versus NG treated RCMVECs (Figure 2B). This suggests that the proteins in GK HG versus NG treated RCMVECs were less similar than the proteins in WKY HG when compared to WKY NG. Further stringent filters, including a *p*-value (*p* < 0.05), were applied to identify the differentially expressed proteins (DEPs) that are significantly altered and sufficiently represented. This resulted in the identification of 26 uniquely altered proteins of the total 214 DEPs (12.1%) in GK NG versus WKY NG RCMVECs. A total of 472 and 274 DEPs were identified in GK HG versus NG treated RCMVECs and WKY HG versus NG treated RCMVECs, respectively (Supplementary Tables S2, S3). Eighty-seven of the 472 DEPs (18.4%) were uniquely altered in GK HG versus NG treated RCMVECs when compared to only 29 of the 274 DEPs (10.6%) in WKY HG versus NG treated RCMVECs (Figure 2C). The complete protein list is detailed in the Supplementary Tables S1–S3.

Filtered proteins were then subjected to pathway analysis using IPA core function. Enrichment and prediction analysis were then performed using the list of DEPs that matched the aforementioned filter criteria. Prediction analysis, which employs *z*-scores to predict the activation/inactivation status of the various pathways and biological functions revealed distinct trends in cardiovascular, metabolic and redox pathways across various comparisons. Under baseline conditions (GK NG versus WKY NG treated RCMVECs), significant differences were mostly restricted to the redox and metabolic processes, while cardiovascular processes were relatively unchanged. Under hyperglycemic states, biological processes involved in cardiovascular health, were predicted to have a reduced functionality in both GK RCMVECs (HG versus NG) and WKY RCMVECs (HG versus NG) (Figure 2D). However, biological functions involved in metabolic processing, such as glycolysis and oxidative phosphorylation (OXPHOS), were predicted to have markedly different activation states in GK HG versus NG treated RCMVECs when compared to WKY HG versus NG treated RCMVECs comparisons (Figure 2D). Based on the protein expression alterations, hyperglycemic conditions were predicted to increase glycolytic activity in WKY RCMVECs (HG versus NG), but reduce it in GK RCMVECs (HG versus NG) in this pathway analysis. On the other hand, hyperglycemic states were predicted to enhance OXPHOS in GK HG versus NG treated RCMVECs, but reduce it in WKY HG versus NG treated RCMVECs. Since glycolytic activity was predicted to be functioning at an enhanced capacity under baseline conditions in GK RCMVECs, the burden of energy production could well be shifted from glycolysis to OXPHOS under hyperglycemic stress.

The DEPs were then subjected to additional filtering using a normalized log2ratio (≥2 or ≤–2), to identify those that were highly enriched. Among the list of filtered DEPs, proteins involved in mediating angiogenic, bioenergetic, metabolic, inflammatory, mitochondrial and oxidation-reduction (redox) functions, are listed in Tables 1–3. This data includes alterations in several metabolic, angiogenic and OXPHOS complex proteins such as glyceraldehyde-3-phosphate dehydrogenase (GAPDH), enolase, cytochrome b, junctional adhesion molecule A (JAM-A) and NADH-ubiquinone oxidoreductase. In the case of GK HG versus GK NG treated RCMVECs (Table 3), reductions in the levels of several metabolic enzymes, including GAPDH (absent, *p*-value < 0.01), aldose reductase (AR; log2ratio = -2.06, *p*-value < 0.01) and beta-enolase (log2ratio = –4.28, *p*-value < 0.01) are indicated. Additionally, there were reductions in pro-angiogenic proteins, such as JAM-A (log2ratio = –5.32, *p*-value < 0.01) and vimentin (unique to GK NG, *p* < 0.01), in GK RCMVECs (HG versus NG; Table 2). Interestingly, a reduction in the levels of pro-angiogenic proteins, such as JAM-A (log2ratio = –4.71, *p*-value < 0.01), were observed in WKY RCMVECs as well (HG versus NG; Table 2), suggesting this may be a direct result of HG independent of the influence of the genetic background. It should also be noted that under baseline conditions, that is GK NG versus WKY NG RCMVECs, GAPDH was detected only in GK NG and not in WKY NG. It could well be that the level of GAPDH detected in GK NG was significantly higher than in WKY NG RCMVECs at baseline prior to hyperglycemic treatment.

### Distinct Metabolic Energy Signature in Vascular Endothelium in Response to Hyperglycemic Conditions and a Permissive Genetic Background for Diabetes

**FIGURE 3:**
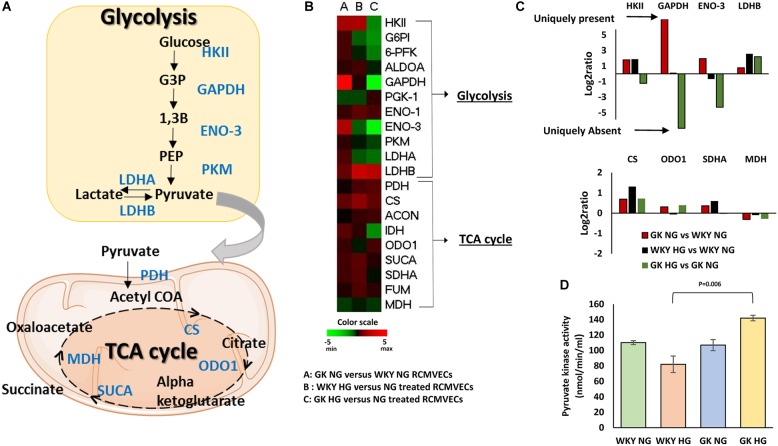
Distinct alterations in endothelial cell metabolism in WKY and GK RCMVECs in response to a hyperglycemic microenvironment. **(A)** Diagrammatic representation of key glycolytic and TCA cycle enzymes detected across the various comparisons. **(B)** Heatmap for the differentially altered enzymes involved in glycolysis and TCA cycle that were detected across various comparisons. **(C)** Magnitude of change of key glycolytic and TCA cycle enzymes across the various comparisons. **(D)** Pyruvate kinase activity assay for WKY and GK (NG and HG) RCMVECs lysates (Values are mean ± SEM; *n* = 3).

Comparison of the expression patterns of enzymes involved in regulation of metabolic functions in endothelial cells revealed distinct trends. Figure 3A represents some of the key glycolytic and tricarboxylic acid (TCA) cycle enzymes that were altered in the study. Under baseline conditions, an upregulation of enzymes involved in both glycolysis and TCA cycle were observed in GK NG when compared to WKY NG RCMVECs (Figure 3B). Hyperglycemic conditions induced changes in metabolic enzymes in GK RCMVECs that were distinctive from those observed in WKY RCMVECs. Compared to their NG treated controls, a noticeable downregulation of enzymes involved in glycolysis was observed in GK RCMVECs under hyperglycemic conditions (Figure 3B). This was not detected in WKY RCMVECs in response to hyperglycemic conditions. Several key glycolytic enzymes including hexokinase-2 (HK II) (log2ratio = –1.23, not significant), GAPDH (unique to GK NG, *p* < 0.01) and enolase-3 (unique to GK NG, *p* < 0.01), were either reduced or undetectable in GK HG versus NG treated GK RCMVECs (Figure 3C and Supplementary Table S3). Lactate dehydrogenase-B (LDH-B), which facilitates a greater availability of pyruvate due to an increased conversion of lactate to pyruvate, was also elevated in both GK and WKY RCMVECs under hyperglycemic conditions. In the case of WKY RCMVECs, hyperglycemic conditions did not significantly alter glycolytic enzymes (Supplementary Table S2). Additionally, expression of several enzymes involved in TCA cycle were slightly increased in both WKY and GK RCMVECS under hyperglycemic conditions (Figure 3C). PKM, which controls the availability of substrates for TCA cycle, was not significantly altered in either proteomic comparisons. PKM is an important enzyme for the metabolic shift that occurs between glycolysis and TCA cycle, since it generates pyruvate, a critical oxidative fuel. Since an increase in TCA cycle activity in endothelial cells has been linked to various diabetic complications, we investigated whether PKM activity is altered in GK and/or WKY RCMVECs under hyperglycemic conditions. Even though protein levels were unchanged, PKM activity was slightly elevated in GK HG versus NG treated RCMVECs (33.9 ± 4.2% increase, not significant), while it was decreased in WKY HG versus NG treated RCMVECs (26.3 ± 12.3% decrease, not significant). However, a significant increase in PKM activity was observed in GK HG RCMVECs was compared to WKY HG RCMVECs (69.6 ± 5.5% decrease, *p* < 0.01) (Figure 3D), suggesting an opposing response based on the background strain.

### Pro-inflammatory and Pro-apoptotic States in the Vascular Endothelium in Response to Hyperglycemic Conditions in the Permissive Diabetes Background

**FIGURE 4:**
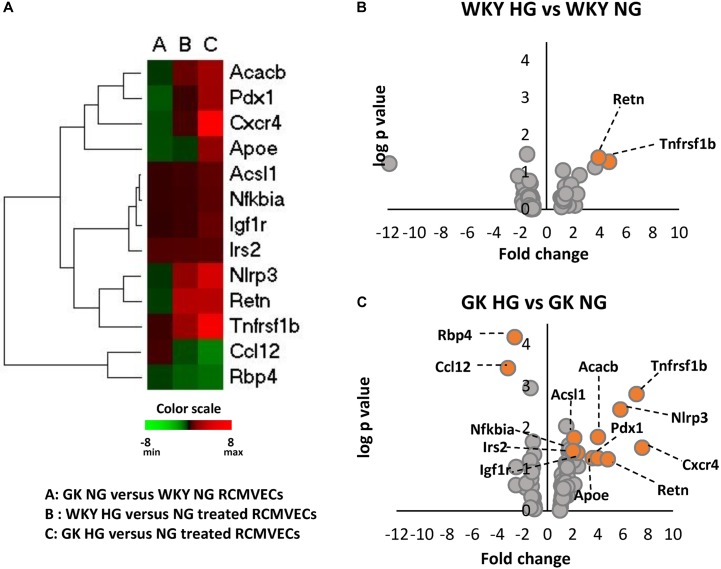
Alterations in the expression of key genes important for metabolic, inflammatory and apoptotic functions in WKY and GK RCMVECs under hyperglycemic conditions. **(A)** Heatmap of 13 significantly altered genes (log2ratio ≤ –2 or ≥ 2 and *p*-value < 0.05) related to metabolic, inflammatory and apoptotic as measured across various comparisons (*n* = 4). **(B)** Volcano plots for differential gene expression between WKY HG versus NG treated RCMVECs. **(C)** Volcano plots for differential gene expression between GK HG versus NG treated RCMVECs. Log *p*-value is plotted on the *y*-axis and fold change is plotted on the *x*-axis. The colored markers denote significantly alteration in the expression of genes (log2ratio of ≤ –2 or ≥ 2, *p*-value < 0.05).

The influence of hyperglycemic conditions on metabolic, inflammatory and apoptotic states in the vascular endothelium in healthy and diabetic states, was assessed by employing a RT-qPCR panel of 84 genes related to insulin resistance and diabetes mellitus (Supplementary Figure S2). Heatmaps and volcano plots were employed to highlight the genes that were significantly altered when filtered for both *p*-value and log2ratio in GK and WKY RCMVECs under HG and NG conditions (*p* < 0.05 and a log2ratio of ≥ 2 or ≤ –2) (Figure 4A–C). In response to HG conditions, the GK RCMVECs, and to a lesser extent the WKY RCMVECs, exhibited significant changes when compared to their respective NG controls. These changes in gene expression include an elevation of genes involved in insulin signaling, adipokine signaling, inflammation, apoptosis and infiltrating leukocyte markers. While two genes were significantly altered in WKY HG versus NG RCMVECs (Figure 4B), 13 genes in total were significantly altered in GK HG versus NG RCMVECs (Figure 4C). Genes involved in inflammation-NACHT, LRR and PYD domains-containing protein 3 (*Nlrp3*, log2ratio = 5.8, *p* < 0.01), insulin signaling- Insulin-like growth factor 1 receptor (*Igf1r*, log2ratio = 2.3, *p* < 0.05), lipid metabolism (*Acsl1*, log2ratio = 2.13, *p* < 0.05), carbohydrate metabolism-Acetyl-CoA carboxylase 2 (*Acacb*, log2ratio = 2.3, *p* < 0.05), apoptosis- Tumor Necrosis Factor Receptor 2 (*Tnfrsf1b*, log2ratio = 7.1, *p* < 0.01) and adipokine signaling- NF-kappa-B inhibitor alpha (*Nfkbia*, log2ratio = 2, *p* < 0.05) were significantly altered in GK HG versus NG treated RCMVECs (Table 4).

**Table 4:** Metabolic, inflammatory and apoptotic gene expression as measured by RT-qPCR in GK and WKY RCMVECs for three comparisons: GK NG versus WKY NG, WKY HG versus WKY NG, GK HG versus WKY HG.

Symbol	GK NG versus WKY NG Log 2 ratio (*p*-value)	WKY HG versus WKY NG Log 2 ratio (*p*-value)	GK HG versus WKY HG Log 2 ratio (*p*-value)	Functions
Acacb	-1.138 (0.046)	2.475 (0.119)	4.025 (0.017)	Carbohydrate metabolism
Acsl1	1.124 (0.973)	1.335 (0.406)	2.133 (0.018)	Carbohydrate metabolism
Apoe	-1.729 (0.389)	-1.230 (0.976)	3.606 (0.054)	Carbohydrate metabolism, metabolite transport
Ccl12	1.437 (0.574)	-1.665 (0.163)	-3.133 (0.0003)	Inflammation
Cxcr4	-1.641 (0.445)	1.535 (0.499)	7.541 (0.030)	Infiltrating leukocyte markers
Igf1r	1.031 (0.705)	1.302 (0.627)	2.389 (0.040)	Insulin signaling
Irs2	1.833 (0.190)	1.840 (0.213)	1.987 (0.039)	Insulin, adipkine signaling; innate immunity and apoptosis
Nfkbia	1.140 (0.997)	1.283 (0.667)	2.033 (0.036)	Adipokine signaling, innate immunity and apoptosis
Nlrp3	-1.088 (0.757)	3.645 (0.072)	5.813 (0.003)	Inflammation, innate immunity and apoptosis
Pdx1	-1.973 (0.230)	1.244 (0.596)	4.008 (0.054)	Non-insulin dependent diabetes mellitus
Rbp4	-1.344 (0.350)	-2.157 (0.126)	-2.599 (0.00006)	Metabolite transport
Retn	-1.292 (0.604)	4.706 (0.049)	4.807 (0.052)	Adipokine signaling
Tnfrsf1b	1.316 (0.467)	3.929 (0.04)	7.091 (0.001)	Adipokine signaling, apoptosis and inflammation


*Gene expression had to meet the following stringency criteria for one of the groups in the comparison (Normalized Log2ratio ≥ 2 or ≤ –2, *p*-value < 0.055) (*N* = 4).*

### Impaired Redox Homeostasis in Vascular Endothelium in Response to Hyperglycemic Conditions and a Permissive Background for Diabetes

**FIGURE 5:**
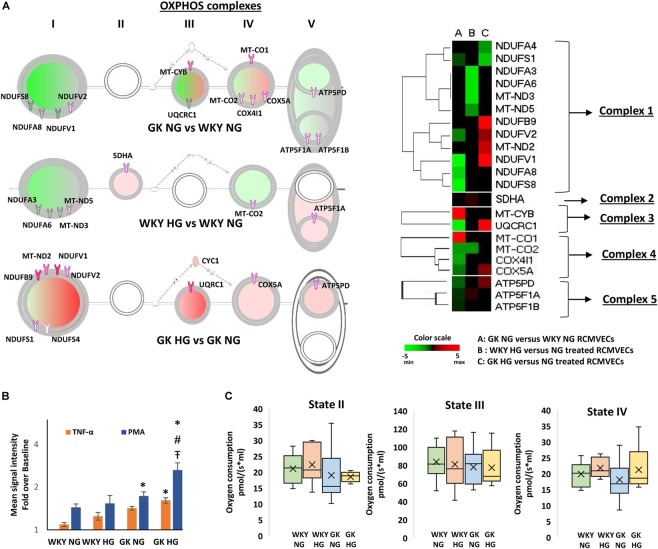
Alterations in OXPHOS signaling pathway expression and function in WKY and GK RCMVECs in response to a hyperglycemic microenvironment. **(A)** IPA schematic representing significant changes in the expression of OXPHOS complex proteins across various comparisons (log2ratio of ≤ –2 or ≥ 2, scan counts ≥ 10 and *p*-value < 0.05). Green shading represents predicted repression, and pink shading represents predicted activation. The intensity of shading denoted the degree of activation or repression. Also shown is the heatmap for the OXPHOS complex proteins that were significantly altered across the various comparisons. **(B)** Significant alterations in ROS production were observed in WKY RCMVECs (NG and HG) and GK RCMVECs (NG and HG) in response to TNF-α or PMA (*n* = 3 biological replicates, 4 technical replicates of each). **(C)** Changes in mitochondrial respiration in WKY RCMVECs [NG (*n* = 8) and HG (*n* = 4)] and GK RCMVECs [NG (*n* = 8) and HG (*n* = 4)] are represented as box and whisker plots. “x” denotes the mean value, while the horizontal line in each of the boxes represents the median. (Values are mean ± SEM). ^∗^ denotes *p* < 0.05 between treated (TNF-α or PMA) and untreated (baseline) samples. 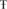 denotes *p* < 0.05 between GK HG or WKY HG compared to their respective controls for the same treatment. # denotes *p* < 0.05 between GK HG and WKY NG for the same treatment.

Pathway analysis identified redox homeostasis as a crucial cellular function to be significantly impaired in GK RCMVECs under hyperglycemic states. The magnitude of oxidative damage is highly dependent on an optimally functioning OXPHOS. Comparison of filtered DEPs [*p*-value (*p* < 0.05), scan counts (≥10) and log2ratio ≥ 2 or ≤ –2], revealed several trends in the expression profiles of OXPHOS complex proteins between GK and WKY RCMVECs, under both hyperglycemic and normal glycemic conditions as shown in the OXHOS schematic and heatmap (Figure 5A). Noticeably, complex I and III were significantly altered. Proteins belonging to complex I were highly downregulated in GK NG versus WKY NG RCMVECs. In the case of complex III, only two proteins were significantly altered. These were mitochondrial encoded cytochrome B (MT-CYB, log2ratio = 4.5) and ubiquinol-cytochrome C reductase core protein (UQRC1, log2ratio = –3.92) (Supplementary Table S1). In GK HG versus NG treated RCMVECs, proteins belonging to complex I were significantly upregulated. Additionally, complex III protein, UQRC1, was also significantly upregulated (log2ratio = 4.21) (Supplementary Table S3). While complex III proteins were relatively unchanged in WKY HG RMVECs, complex I proteins were also downregulated in WKY HG versus NG treated RCMVECs (Figure 5A).

Since we observed differential protein expression patterns between GK and WKY RCMVECs related to the mitochondrial OXPHOS complexes that are linked to ROS production, when subjected to hyperglycemic conditions, we tested whether the cells would exhibit a prominent difference in ROS production in response to stress stimuli. Using the CellROX green reagent we were able to observe a weak baseline production of ROS in both the WKY and GK RCMVECs subjected to both normal and hyperglycemic conditions (Supplementary Figure S3). In order to look at the stress response in the groups, these baselines were normalized and the degree of the ROS formation in relation to cell stress was quantified. When the RCMVEC groups were subjected to known ROS triggers, TNF-α (500 ng/mL) or PMA (10 Um), amplification of oxidative stress response was observed in both WKY and GK RCMVECs, under both conditions (Figure 5B). The most striking augmentation was observed in PMA-treated GK RCMVECs under hyperglycemic conditions. The magnitude of ROS production was significant in PMA-treated GK RCMVECs under HG and NG conditions. TNF-α treatment caused a significant spike in ROS production in only GK HG RCMVECs. This data suggests an impaired ability to regulate stress-induced ROS production and accumulation in the GK background, under normal and hyperglycemic conditions, to a greater extent than in the WKY endothelium. This could be indicative of an impaired redox machinery in response to stress stimuli.

Mitochondrial respiration experiments, which serve as a direct measure of mitochondrial bioenergetics, were carried out on intact isolated mitochondria from GK and WKY RCMVECs under NG and HG conditions. State II respiration, measured in the presence of substrates (glutamate and malate) without the addition of ADP, was not significantly altered either by the genotype or by subjecting the cells to hyperglycemic conditions. While ADP-stimulated respiration (state III) was also not significantly different across the different comparisons, it was observed to be lower in GK RCMVECs under both normal glycemic and hyperglycemic conditions (Figure 5C). Additionally, there was a non-significant increase in state IV respiration, the respiration rate following the complete conversion of ADP to ATP, in both WKY HG and GK HG RCMVECs when compared to their respective normal glycemic controls (Figure 5C).

### Impaired Angiogenic Capacity in Vascular Endothelium in Response to Hyperglycemic Conditions and a Permissive Background for Diabetes

**FIGURE 6:**
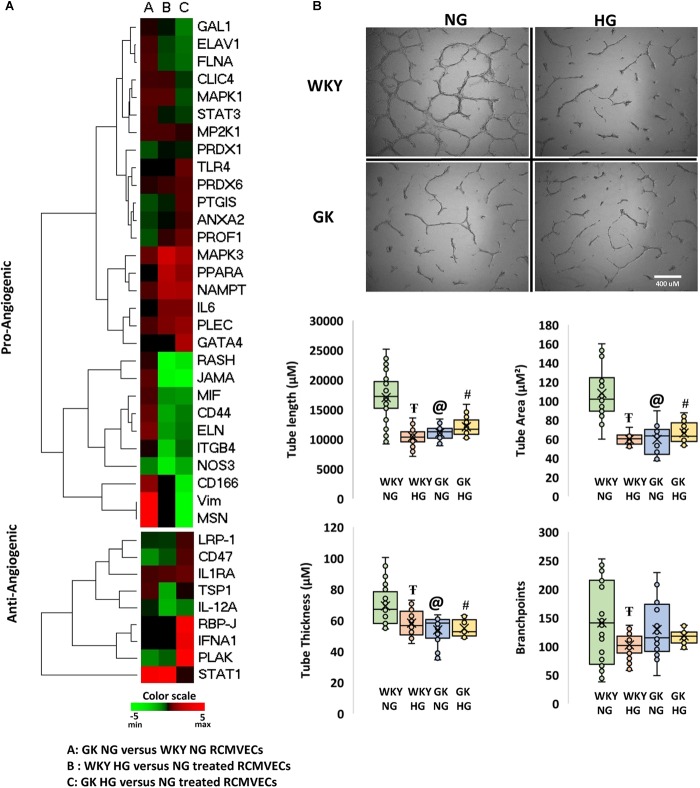
Characterization of the angiogenic capability of WKY and GK RCMVECs in a hyperglycemic microenvironment: **(A)** Heatmap denoting the various pro- and anti- angiogenic protein expression for across the various comparisons. **(B)** The function comparison of the RCMVECs under varying conditions was measured by tube formation parameters. Tube length, tube area, tube thickness and branchpoints were all measured for WKY RCMVECs (NG and HG) and GK RCMVECs (NG and HG). Values are mean ± SEM (*n* = 3; 8 technical replicates of each). T denotes *p* < 0.05 between GK HG or WKY HG compared to their respective normal glycemic controls. @ denotes *p* < 0.05 between GK NG and WKY NG). # denotes *p* < 0.05 between GK HG and WKY NG.

Since both hyperglycemic conditions and diabetic states have been established to impair angiogenesis, we investigated the angiogenic potential of the GK and WKY RCMVECs under HG and NG conditions in culture. Apart from vimentin and moesin, which were both uniquely detected in GK NG when compared to WKY NG RCMVECs, no marked changes were observed in the pro-angiogenic protein expression for the baseline comparison. Several pro-angiogenic proteins such as JAM-A (WKY HG versus NG: log2ratio = –4.71, *p* < 0.01; GK HG versus NG: log2ratio = –5.32, *p* < 0.01), filamins (FLNA, WKY HG versus NG: log2ratio = –0.47, *p* < 0.01; GK HG versus NG: log2ratio = –0.88, *p* < 0.01) and HRas (RASH, WKY HG versus NG: unique to NG, *p* < 0.01; GK HG versus NG: unique to NG, *p* < 0.01) were downregulated, while nicotinamide phosphoribosyltransferase (NAMPT, WKY HG versus NG: log2ratio = 2.07, not significant; GK HG versus NG: log2ratio = 1.97, *p* < 0.01) was upregulated in both GK and WKY RCMVECs under hyperglycemic states (Figure 6A). NAMPT has been previously observed to elevated under high glucose conditions since its function is to extend the duration of cell survival under hyperglycemic stress (Borradaile and Pickering, 2009). However, distinctive differences could be observed in anti-angiogenic protein expression in the GK HG versus NG RCMVECs as compared to WKY HG versus NG RCMVECs. Compared to WKY RCMVECs, a noticeable upregulation of anti-angiogenic proteins, such as junction plakoglobin (PLAK), CD47 and Interferon-α (IFNA) were observed in GK RCMVECs under hyperglycemic conditions. In order to assess the angiogenic capacity in WKY and GK RCMVECs under both normal and hyperglycemic states we utilized TFA. Alterations in tube formation were measured using several parameters to assess functions including tube length, area, thickness and branchpoints (Figure 6B). WKY HG versus NG treated RCMVECs displayed a significant reduction in tube length (34.9 ± 1.9% reduction, *p* < 0.05), area (44.5 ± 1.1% decrease, *p* < 0.05) and thickness (15.9 ± 2.5% reduction, *p* < 0.05). Under baseline conditions (normal glucose), significant impairment in the ability of RCMVECs to form tubes was also observed in GK NG RCMVECs when compared to WKY NG RCMVECs as measured by a reduction in tube length (38.6 ± 2.2% reduction, *p* < 0.01), area (44.8 ± 2.6% decrease, *p* < 0.01) and thickness (22.3 ± 2.9% reduction, *p* < 0.01) (Figure 6B). However, GK HG had shown no further enhancement of the impaired phenotype versus GK NG RCMVECs, suggesting that GK RCMVECs have additional underlying mechanisms of angiogenic impairments as compared to WKY RCMVECs that require hyperglycemic microenvironment for impairments to occur. This data also suggests that there are baseline differences in the function and angiogenic potential of WKY and GK endothelial cells under normal glycemic conditions, suggesting there is potentially a genetic component that is inherently different in the endothelial cells derived from the control and diabetes models that is retained in culture.

### Additional Targets of Interest Derived From the Dataset

In addition to the protein pathways and correlated functional outputs already discusses, there are other key targets or pathways that may play an important role in the endothelial response to hyperglycemia in states of diabetes. While NAMPT was discussed above in relation to the potential angiogenic influence, this target is also potentially an important metabolism regulatory protein. Interestingly, NAMPT was upregulated in response to HG in both the WKY (log2ratio = 2.07, not significant) and the GK (log2ratio = 1.97, *p* < 0.01) RCMVECs under hyperglycemic states as compared to the NG counterparts (Figure 6A). NAMPT has been previously observed to elevated under high glucose conditions since its function is to extend the duration of cell survival under hyperglycemic stress (Borradaile and Pickering, 2009). NAMPT also acts as a key regulator of SIRT1, the latter being a key protein involved in maintaining homeostasis and offering protection again metabolic abnormalities (Imai and Yoshino, 2013; Satoh et al., 2013). Interestingly this compensatory mechanism was retained in both cell types in response to hyperglycemia.

While it is well known that inflammation is often at an elevated imbalance during states of hyperglycemia and diabetes, there were some interesting targets that can be pursue in the dataset provided here for future studies. One such protein of interest is the CD59 glycoprotein that limits damage caused by the activation of the immune system and autoimmunity (Motoyama et al., 1992; Alegretti et al., 2012). CD59 was down regulated in the WKY (log2ratio = –3.86, *p* < 0.01) and GK (log2ratio = –3.63, *p* < 0.01) RCMVECs in response to hyperglycemic conditions (Tables 2, 3). It has also been shown that both a deficiency and glycosylation of CD59 can lead to functional impairment and an autoimmunity response (Qin et al., 2004; Nevo et al., 2013), which may warrant future consideration in relation to hyperglycemia during diabetic states.

There were also noticeable differences in Acyl-CoA synthetases, which have key roles in the beta-oxidation of fatty acids. Oxidation of fatty acids is deemed as a vital source of elevated mitochondrial ROS so this may be an avenue to pursue if overactive. An increase in five enzymes involved in fatty acid oxidation was observed in GK HG versus NG RCMVECs, including long-chain fatty acid-CoA ligase 5 (HG only, *p* = 9.4 E-20), 3-ketoacyl-CoA thiolase (log2ratio = 3.32, *p* = 5.8E-05), Medium-chain specific acyl-CoA dehydrogenase (log2ratio = 2.96, *p* = 0.0005), non-specific lipid-transfer protein (log2ratio = 2.62, *p* = 7.6E-34), and peroxisomal multifunctional enzyme type 2 (log2ratio = 2.27, *p* = 2.1E-15). Only non-specific lipid-transfer protein (log2ratio = 2.25, *p* = 2.1E-17) and peroxisomal multifunctional enzyme type 2 (log2ratio = 2.68, *p* = 7.5E-19) were significantly elevated in WKY HG versus NG RCMVECs. While the targets noted here are examples of potential avenues to pursue mechanistically and an exhaustive set can be found in the Supplementary Tables S1–S3 with the complete protein comparison data.

## Discussion

**FIGURE 7:**
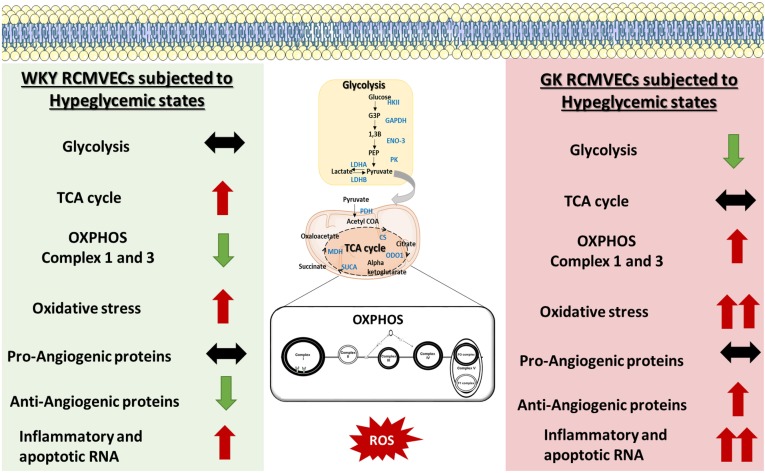
Schematic of the molecular mechanisms altered by hyperglycemic states in GK RCMVECs and WKY RCMVECs. GK RCMVECs subjected to a hyperglycemic microenvironment resulted in changes that are distinct to those observed in WKY RCMVECs under high glucose states. These include differences in glycolytic enzyme expression, expression of OXPHOS complex 1 and 3 proteins, anti-angiogenic protein expression and inflammatory and apoptotic RNA expression. Red arrows indicate upregulation or predicted activation (↑), green arrows indicate downregulation or predicted repression (↓), black arrows indicates a lack of prominent upregulation or downregulation (**↔**). A greater degree of change is denoted by double arrows (↑↑, ↓↓).

Through the integration of high-throughput tandem LC-MS/MS analysis, transcriptomic analysis, bioinformatics analyses, and *in vitro* functional assays, we were able to delineate a wide spectrum of molecular derangements in endothelial cells that were triggered by environmental (hyperglycemic) and/or genetic (diabetic permissive) determinants. While we identified several differentially abundant proteins in RCMVECs derived from GK and WKY rats under normal glycemic conditions (Supplementary Table S1 for the complete list), the major finding was the identification of differential protein and gene expression in GK HG versus NG treated when compared to that observed in WKY HG versus NG treated RCMVECs. The distinctively altered targets of hyperglycemic stress were identified to play a key role in elevating oxidative stress, and diminishing key metabolic functions in the endothelium. The derangements in redox and angiogenic machinery was further validated by employing *in vitro* functional assays. A comparison of the differences and similarities in mechanistic alterations triggered by hyperglycemia in GK and WKY RCMVECs is summarized via a schematic in Figure 7. This study also provides a large, comprehensive resource for other researchers in the field of the study that are interested in the molecular mechanisms of hyperglycemia-induced vascular damage in diabetic and non-diabetic states (Supplementary Tables S1–S3). While the current study is limited in that the altered endothelial phenotypes were correlated with the dataset, this data provided here will act as a comprehensive resource of expression in the WKY and GK endothelium under varying glycemic conditions can lead to multiple avenues of mechanistic testing. It is important to note that most studies in the literature focus on the dysfunctional diabetic vascular endothelium, while there is limited focus on how the healthy or the diabetic endothelium responds to a hyperglycemic environment. This study will help delineate differences in the endothelial response to the hyperglycemic microenvironment and provide snapshots into the molecular mechanisms that occur at different stages during the onset and progression of diabetes.

A quantitative and a qualitative comparison performed on the DEPs in GK and WKY RCMVECs under hyperglycemic and normal conditions, revealed several noticeable trends. As the percentage of DEPs was greater in GK HG versus NG treated RCMVECs, when compared to WKY HG versus NG treated RCMVECs, we can infer that hyperglycemic conditions were able to alter a greater number of proteins in a diseased endothelium than a healthy endothelium. Qualitative assessment, by way of pathway analysis, of the DEPs resulted in the prediction of a greater impact of hyperglycemic states on several biological processes associated with endothelial dysfunction in GK RCMVECs as compared to the response in WKY RCMVECs. These alterations in the GK RCMVECs includes a potential impairment in glycolytic enzymes, an increase in oxidative stress, and a decrease in angiogenic potential (Figure 2D). Several groups have reported an increased susceptibility of endothelial cells under a diabetic milieu to toxic hyperglycemic insults (Renard et al., 2004; Azcutia et al., 2010; Zitman-Gal et al., 2010). Our data here supports this and adds another layer to it by defining many key molecular differences behind how the susceptible diabetic endothelium responds to the hyperglycemic condition versus the healthy, non-diabetic endothelium.

One of the hallmarks of endothelial cell function, is their ability to shift to a pro-angiogenic phenotype in response to environmental cues. By the same token, prolonged hyperglycemic stress can hinder endothelial cells from assuming an angiogenic phenotype (Prisco et al., 2014). Subjecting RCMVECs from GK and WKY rats to a hyperglycemic environment, resulted in an altered expression pattern of angiogenic proteins. Several pro-angiogenic proteins were downregulated in both GK and WKY RCMVECs under hyperglycemic environment. Proteins involved in maintaining endothelial barrier function, such as JAMs (Wallez and Huber, 2008); proteins which are essential for vascular, growth and development, such as FLNA (Feng et al., 2006; Del Valle-Pérez et al., 2010) and proteins that are key regulators of VEGF-induced angiogenesis, such as RASH (Serban et al., 2008) were significantly decreased in both GK and WKY RCMVECs under hyperglycemic conditions (Figure 6A and Supplementary Tables S2, S3). Additionally, CD59, a glycoprotein that limits damage caused by the activation of complement system (Motoyama et al., 1992; Alegretti et al., 2012), was decreased in both GK and WKY RCMVECs under hyperglycemic conditions (Tables 2, 3). The CD59 glycoprotein aids in preventing activation of the immune system and autoimmunity (Motoyama et al., 1992; Alegretti et al., 2012) and when this protein is either deficient or increased in glycosylation it can no longer block this autoimmune response (Qin et al., 2004; Nevo et al., 2013), which may warrant future consideration in relation to hyperglycemia and tissue degradation during progressive diabetic states. Our results also suggests that hyperglycemic states alone can impair endothelial function and angiogenesis. However, hyperglycemic states also induced distinctive changes in GK RCMVECs. Moesin, a downstream regulator of urokinase receptor-mediated cell adhesion and angiogenesis (Degryse et al., 2017), was downregulated (uniquely detected in NG, *p* < 0.01) under hyperglycemic conditions in GK RCMVECs as compared to the normal glycemic control (Table 3). Interestingly, moesin was also uniquely detected in GK RCMVECs, and not in WKY RCMVECs, suggesting that the expression in GK RCMVECs was significantly greater than in WKY RCMVECs (Table 1). GK RCMVECs, but not WKY RCMVECs, subjected to hyperglycemic conditions resulted in a pronounced increase in the levels of anti-angiogenic proteins (Figure 6A). Anti-angiogenic factors such as PLAK (Nagashima et al., 1997), CD47 (Kaur and Roberts, 2011) and IFNA (Indraccolo, 2010), were observed to be upregulated in hyperglycemic GK RCMVECs (Figure 6A and Supplementary Tables S2, S3).

Although we observed a pronounced increase in some anti-angiogenic proteins in GK HG versus NG treated RCMVECs, a decrease in pro-angiogenic proteins in both GK and WKY under hyperglycemic conditions suggested that hyperglycemia may well diminish angiogenesis independent of a genetic predisposition. In order to evaluate differences in angiogenic potential between GK and WKY RCMVECs, under hyperglycemic and normal glycemic conditions, functional TFA experiments were carried out. While a significant decrease in several TFA parameters (length, area and branchpoints) were observed in WKY cells subjected to hyperglycemic states versus the normal glycemic control, glycemic environment appeared to have no effect on the already impaired GK RCMVECs (Figure 6B). Significant deficiencies in the ability of GK RCMVECs to form robust tubes when compared to WKY RCMVECs in normal glycemic baseline conditions, highlights a defective angiogenic machinery in the GK endothelial cells under diabetic states regardless of glycemic microenvironment. The inherent defects in GK RCMVECs resulting in fractured tubes could possibly offset any additional negative effects that hyperglycemia alone may have on the angiogenic ability of GK RCMVECs, as they are already significantly impaired. As WKY RCMVECs, the non-diabetic endothelium, exhibited a greater susceptibility to a high glucose environment, we also infer that hyperglycemic states alone can induce significant alterations in the angiogenic functionality of endothelial cells. This is in accordance with several preclinical and clinical studies that have reported the harmful effects of hyperglycemia alone on healthy endothelium (Williams et al., 1998; Beckman et al., 2001; Prisco et al., 2014).

Metabolic pathways and redox homeostasis, fundamental for endothelial cell survival, proliferation and function, have been reported to be altered under hyperglycemic environments (Aronson and Rayfield, 2002; Campos, 2012). In our study, we investigated whether hyperglycemic stress alters metabolic, inflammatory and redox processes to the same extent in a healthy versus a diseased endothelium. Hyperglycemic conditions resulted in significant distinctive alterations in the expression of metabolic, inflammatory and redox genes in GK when compared to that of WKY RCMVECs. The expression of genes involved in insulin signaling (*Igf1r, Irs2*) and inflammatory conditions (*Cxcr4, Nlrp3)* was elevated in GK RCMVECs, but not WKY RCMVECs under hyperglycemic conditions. Elevation in pro-inflammatory states is a crucial factor in the disruption of vascular homeostasis in diabetic states (Tabit et al., 2010). *Acsl1*, which has been reported to exacerbate fatty acid-induced apoptosis in endothelial cells (Li et al., 2013), was significantly elevated in GK HG versus NG treated RCMVECs only. Additionally, a greater fold change was observed in the pro-apoptotic gene, *Tnfrsf1b*, in GK HG RCMVECs when compared to WKY HG RCMVECs. (WKY HG versus NG, log2ratio = 3.929, *p* = 0.040; GK HG versus NG, log2ratio = 7.091, *p* = 0.001). This suggests that hyperglycemic states is able to exacerbate inflammatory and apoptotic states more so in a diseased endothelium than in a healthy one (Figure 4A–D and Table 3).

Metabolic processes, such as glycolysis, are crucial for endothelial cell survival and proliferation. Our proteomic data revealed a global downregulation of glycolytic enzymes in GK HG RCMVECs, but not WKY HG RCMVECs (Figure 3B and Supplementary Tables S2, S3). Critical glycolytic enzymes, such as the rate limiting enzyme HK II, beta enolase and GAPDH were detected to be downregulated in the GK HG versus NG treated RCMVECs (Figure 3B,C and Supplementary Table S3). This suggests that endothelial glycolytic processes may be diminished in a diseased endothelium but may be normally functional in a healthy endothelium following hyperglycemic exposure. Dampening of GAPDH activity has been demonstrated as a critical step for hyperglycemia-induced vascular damage (Du et al., 2003). Suppression of glycolysis results in a metabolic shift from glycolysis to alternative bioenergetics pathways such as the pentose phosphate pathway (PPP) and OXPHOS (Lee and Yoon, 2015; Kathagen-Buhmann et al., 2016). Since the rate limiting enzyme of PPP, Glucose-6-phosphate dehydrogenase, is also decreased in GK HG versus NG treated RCMVECs (log2ratio: –1.1, *p* < 0.05; Supplementary Table S3), OXPHOS could well be the major bioenergetics pathway in GK RCMVECs under hyperglycemic stress. PKM, the enzyme that bridges glycolysis to the TCA cycle, was observed to have the greatest activity in GK HG RCMVECs (Figure 3D). Since pyruvate is a major oxidative fuel and a critical substrate for driving TCA cycle, a metabolic shift to OXPHOS could be favored in endothelial cells under hyperglycemic stress in diabetic states.

Although OXPHOS results in a greater ATP turnover, endothelial cells are well-documented to rely heavily on glycolysis to meet their quick energy demand (Culic et al., 1997). A major reason being that high dependence on OXPHOS leads to an augmentation in the levels of ROS, and thereby endothelial cell damage (Dey and Moraes, 2000; Hüttemann et al., 2008; Liemburg-Apers et al., 2015). Mitochondrial respiration complexes I and III proteins of the OXPHOS pathways are considered to be major sites of mitochondria-generated ROS in the cell (Chen et al., 2003). Proteins specific to both complex I and III were detected at elevated levels in GK HG RCMVECs versus the NG counterpart (Figure 5A); an effect not observed in the WKY HG versus NG treated RCMVECs. This coupled with a decrease in the levels of proteins involved in mitochondrial stability, such as protein QIL1 (uniquely present in NG, *p* < 0.01; Supplementary Table S2) in GK HG versus NG treated RCMVECs, signifies a disruption in redox homeostasis (Guarani et al., 2015). By employing ROS inducers such as TNF-α and PMA, we observed a greater ROS production in GK RCMVECs, when compared to WKY RCMVECs, under hyperglycemic and normal glycemic conditions. PMA and TNF-α are well-known to increase ROS levels through mitochondrial and non-mitochondrial mechanisms, such as disruption of mitochondrial membrane potential (Kim et al., 2010) and NOX2 activation (Cox et al., 1985). In fact, dysfunctional endothelial mitochondria has been reported to severely diminish angiogenic ability and cell survival (Pangare and Makino, 2012). Although mitochondrial respiration, which serves as a direct assessment of mitochondrial functions, was reported by others to be diminished under diabetic conditions (Mogensen et al., 2007; Phielix et al., 2008), our data suggests a minimum impact of diabetic states and/or hyperglycemic states on it in RCMVECs. While we did observe a combination of reduced state III and an elevated state IV respiration in GK HG versus NG treated RCMVECs, which is indicative of reduced capacity for substrate oxidation and ATP production, the changes were not statistically significant. It should be noted that hyperglycemic states were reported to impact mitochondrial respiration in kidney cells, but not in cardiac cells (Lashin and Romani, 2004; Czajka and Malik, 2016). It could well be that mitochondrial respiration is dependent on both the severity of hyperglycemic conditions, and the cell type (Lashin and Romani, 2004; Rabøl et al., 2009), along with the duration of exposure.

Considering the above mechanistic changes, we conclude that a combination of hyperglycemic environmental insult and a diabetes permissive background leads to an array of protein and gene expression changes that are distinct from those that are due to hyperglycemia alone. However, it should be noted that these datasets are correlative in nature and act as a resource for researchers in the field to perform future mechanistic studies on differential targets. These future mechanistic validations will be very important, as with any proteomics data there can be dynamic exclusion issues with the data collection that can influence the expression values obtained and there can be distinct feedback mechanism in transcriptomic data that may not correlate well with the protein present. As such it is often valuable to approach these datasets from a pathway perspective in addition to looking at individual expression components. This study also focused on WKY and GK RCMVECs and the response seen in additional rat models or endothelial cell types still need to be explored to provide data with enhanced genetic diversity. To translate these studies future validation work will also need to be done in animal models to move beyond the limitations that can sometimes occur with studies in cell culture.

Regardless of these limitations, this study provides a comprehensive molecular and phenotypic resource to open opportunities for future mechanistic validation studies in the WKY and GK endothelium, as well as in other model systems. The differences that we have seen between the response of the normal WKY endothelium and diabetic GK endothelium in response to hyperglycemic insult is of interest in itself because it allows exploration of the environmental versus genetic influences. A combination of functional *in vitro* experiments and bioinformatics analyses of the datasets confirmed the deleterious impact of both genetic (diabetic) and environmental (hyperglycemic) factors on the vascular endothelium. The data from this study had shown compromised insulin and angiogenic signaling in combination with an elevated superoxide burden, possibly due to an increased OXPHOS activity. From the data we infer that the consequence of an incapacitated glycolytic machinery leads to a greater reliance on the OXPHOS pathway in GK HG RCMVECs. This may eventually result in a significant superoxide burden, leading to a spike in the levels of pro-atherogenic factors. Derangements in the endothelial glycolytic machinery leading to a metabolic shift toward OXPHOS could be detrimental, since it has been demonstrated to lead to an inefficient vessel sprouting and proliferation (De Bock et al., 2013). Since the exposure of the susceptible endothelium to hyperglycemic stress leads to a significant rewiring of homeostatic biological processes, the ability of the cell to function in sensory and effector capacities may also be significantly hindered. Future studies should be aimed at understanding the significance of alteration of various molecules involved in metabolism and superoxide production in endothelial cells under both susceptible genetic background and hyperglycemic microenvironments. These pathways are candidate mechanisms by which hyperglycemia may induce significant vascular complications in diabetic states and the major findings are summarized in Figure 7.

The results of the current study also provide new leads for the identification of novel biomarkers and/or therapeutic targets for vascular endothelial dysfunction as related to a varying states of glycemic control. Elevated levels of components of the inflammatory and immune response were also observed (Supplementary Tables S1–S3) and should be pursued in future mechanistic studies for possible contributions to tissue damage in progressive diabetes. For instance, inherent differences in CD59, as detected here, could have a major impact on the autoimmunity response and tissue degradation during progressive diabetes (Qin et al., 2004; Nevo et al., 2013). Glycosylation of CD59 also prevents the protein from functioning properly (Qin et al., 2004; Nevo et al., 2013) and this may be an important overlooked route of dysfunction. Moving forward it will be important to not only look at protein or gene changes, but to also look at changes in protein glycosylation that could alter function during hyperglycemic states. Increases in spontaneous glycosylation through Maillard reactions is a much understudied area and could be extremely valuable moving forward with studies aimed at understanding molecular changes during hyperglycemic states. Pursuing these future avenues will be fundamental in designing new and effective therapeutic strategies that have the potential to counter the progression of tissue and organ damage in diabetes.

## Ethics Statement

This study was carried out in accordance with the recommendations of the Medical College of Wisconsin Institutional Animal Care and Use Committee (IACUC) and Institutional Biosafety Committee (IBC) in accordance with the guidelines established by the National Institutes of Health. All protocols were approved by the Medical College of Wisconsin IACUC and IBC prior to the study. Rat models used were commercially purchased from Taconic Biosciences and/or Charles River for research purposes. The rat models were all properly cared for in our well-controlled Biomedical Animal Resource Center at the Medical College of Wisconsin.

## Author Contributions

BH and DH contributed to all aspects of the project including experimental planning/conceptualizing, experimentation, data analysis, and manuscript writing and editing. TM, AV, and NT contributed to experimentation, data analysis, and manuscript editing. RD contributed to experimental planning/conceptualizing and manuscript editing.

## Conflict of Interest Statement

The authors declare that the research was conducted in the absence of any commercial or financial relationships that could be construed as a potential conflict of interest.

## Footnotes

1 www.ingenuity.com
