# Supplementary material for: Influence of a Hyperglycemic Microenvironment on a Diabetic Versus Healthy Rat Vascular Endothelium Reveals Distinguishable Mechanistic and Phenotypic Responses
Source: Front Physiol. 2019 May 10;10:558. doi: 10.3389/fphys.2019.00558 (PMC6524400; doi:10.3389/fphys.2019.00558)
Supplement: Supplementary file 1 [file Data_Sheet_1.PDF]

Supplementary Figures

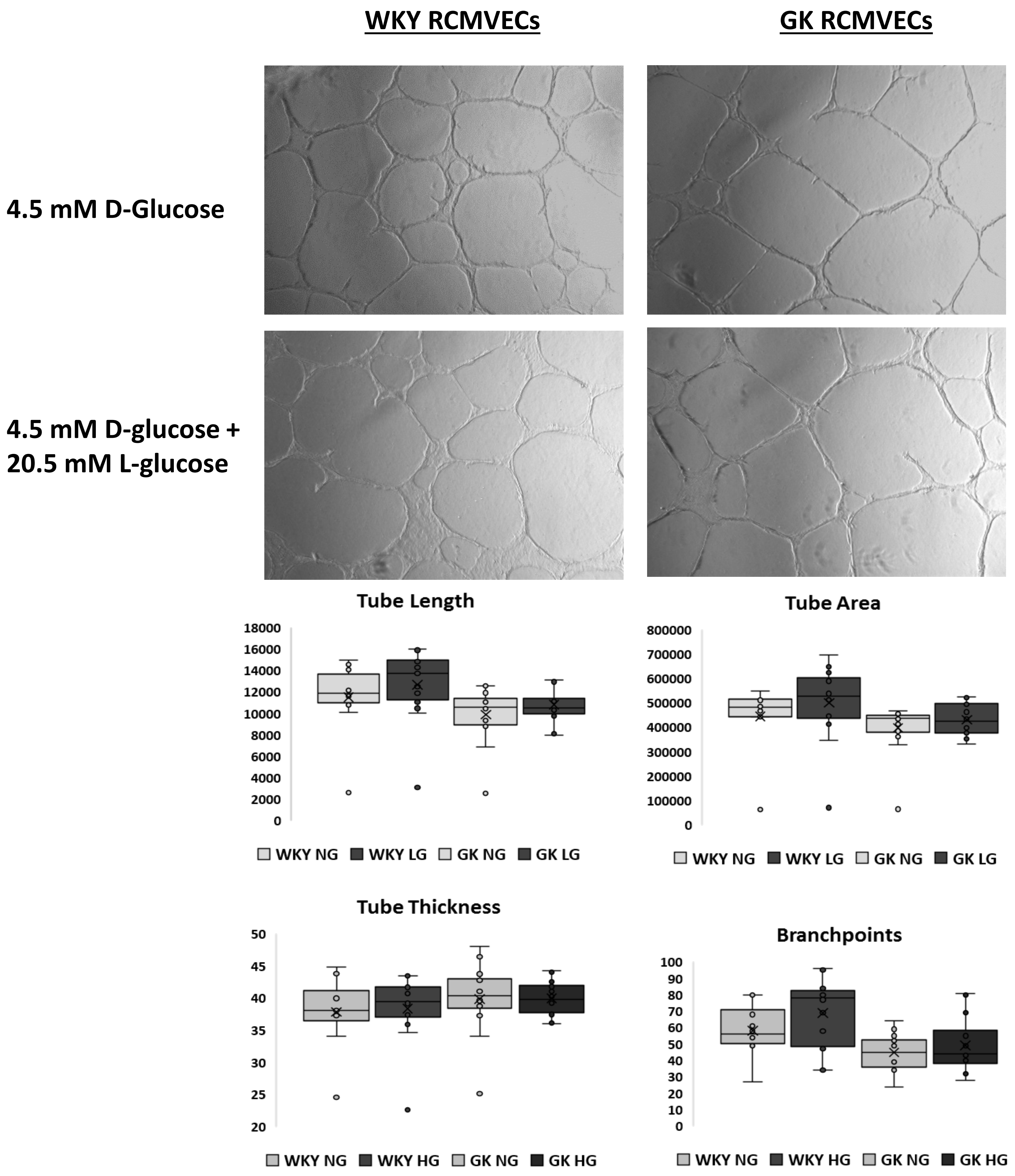

Supplementary Figure 1. The hyperosmotic effect of the media with supplemented glucose on the Wistar Kyoto (WKY) and Goto-Kakizaki (GK) rat cardiac microvascular endothelial cells (RCMVECs) was tested to ensure the differences observed with 25 mM D-glucose (active form utilized) was responsible and not the simple increase in osmolarity. To test this, WKY and GK RMCMVECs were subject to a tube formation assay following a week and a half treatment with the either MCDB131 EGM-MV media with 4.5 mM D-glucose (normal glucose media) or 4.5 mM D-glucose plus 20.5 mM L-glucose (inactive form not utilized) bringing the media to 25 mM total. No difference was observed between the normal and high glucose media supplemented with L-glucose suggesting this is not inhibiting the function in our assay (N=5).

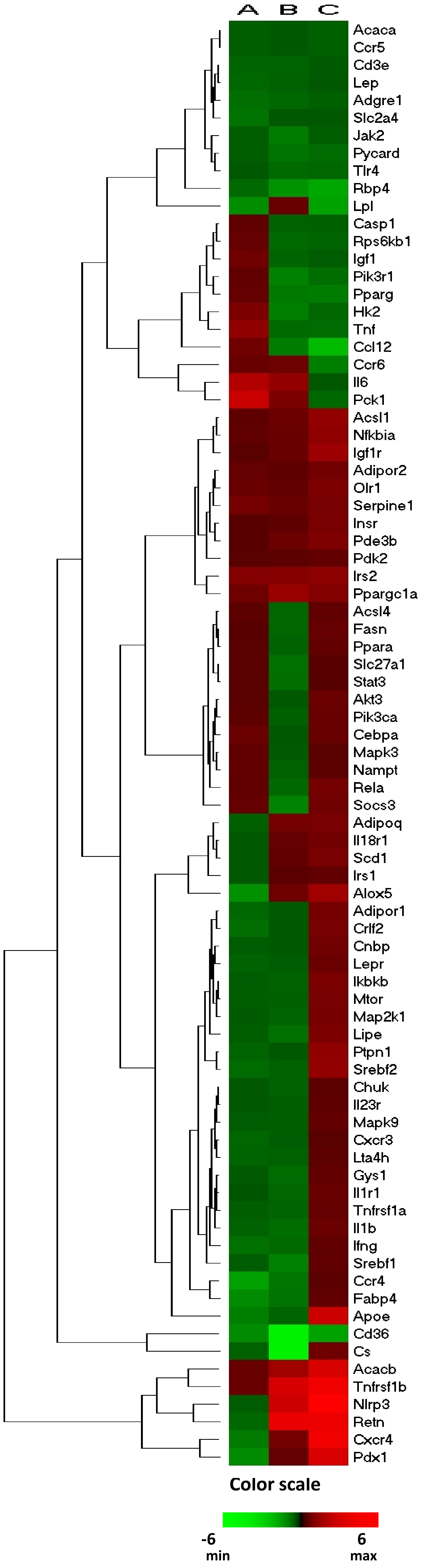

Supplementary Figure 2. Displayed is a heatmap representing the expression of 84 genes involved in biological processes affected related to insulin signaling and diabetes. The three columns represent (1) GK normal glucose (NG; 4.5 mM) versus WKY NG; (2) WKY high glucose (HG; 25 mM) versus NG treated RCMVECs; (3) GK HG vs NG treated RCMVECs.

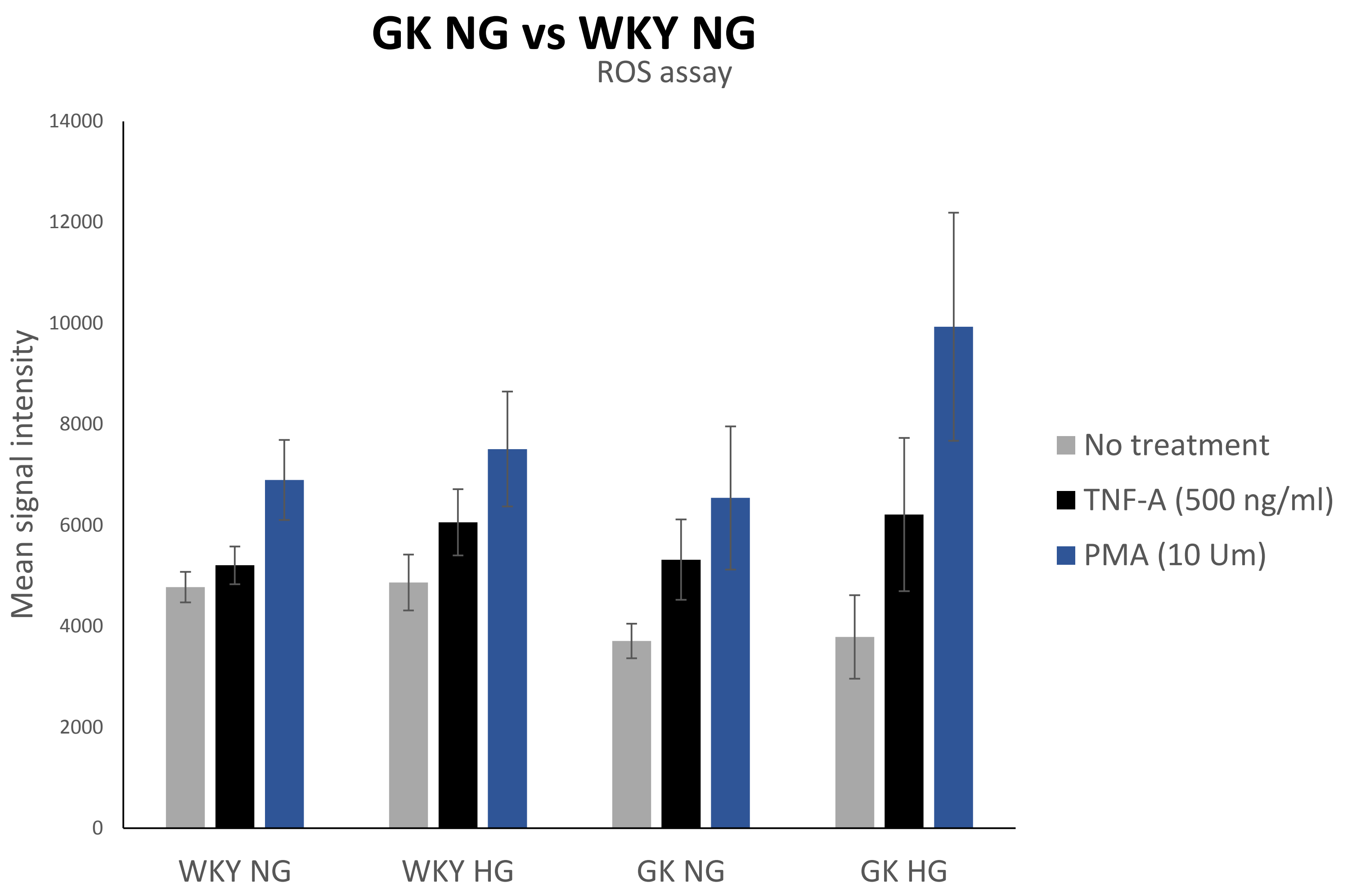

Supplementary Figure 3. Heatmap representing the expression of 84 genes involved in biological processes affected in diabetic and hyperglycemic conditions. The three columns represent (A) GK NG vs WKY NG; (B) WKY HG vs NG treated RCMVECs; (C) GK HG vs NG treated RCMVECs. N=3
